# Glycopeptide Resistance in Gram-Positive Cocci: A Review

**DOI:** 10.1155/2012/781679

**Published:** 2012-06-19

**Authors:** S. Sujatha, Ira Praharaj

**Affiliations:** Department of Microbiology, Jawaharlal Institute of Postgraduate Medical Education and Research (JIPMER), Puducherry 605006, India

## Abstract

Vancomycin-resistant enterococci (VRE) have emerged as important nosocomial pathogens in the past two decades all over the world and have seriously limited the choices available to clinicians for treating infections caused by these agents. Methicillin-resistant *Staphylococcus aureus*, perhaps the most notorious among the nosocomial pathogens, was till recently susceptible to vancomycin and the other glycopeptides. Emergence of vancomycin nonsusceptible strains of *S. aureus* has led to a worrisome scenario where the options available for treating serious infections due to these organisms are very limited and not well evaluated. Vancomycin resistance in clinically significant isolates of coagulase-negative staphylococci is also on the rise in many setups. This paper aims to highlight the genetic basis of vancomycin resistance in *Enterococcus* species and *S. aureus*. It also focuses on important considerations in detection of vancomycin resistance in these gram-positive bacteria. The problem of glycopeptide resistance in clinical isolates of coagulase-negative staphylococci and the phenomenon of vancomycin tolerance seen in some strains of *Streptococcus pneumoniae* has also been discussed. Finally, therapeutic options available and being developed against these pathogens have also found a mention.

## 1. Introduction

Vancomycin was the first glycopeptide antibiotic to be discovered as early as 1950 [1]. However, its toxicity profile and the availability of less toxic alternatives like the beta-lactams made its use for gram-positive infections quite rare. It was only after the large-scale emergence and spread of methicillin-resistant* S. aureus* (MRSA) strains and extensive beta-lactam resistance that this agent gained prominence.

And it was not until 30 years later that the first clinical isolates with reduced susceptibility to vancomycin were described. Vancomycin resistance was first described in isolates of *Staphylococcus epidermidis* [1]. Vancomycin resistance in enterococci was first described in Europe in the late 1980s and spread to much of the developing world. The first isolate of *S*. *aureus* with reduced susceptibility to vancomycin was reported from Japan in 1997 and had a vancomycin MIC in the intermediate susceptibility range [2].

Although primary vancomycin resistance has been described in many bacterial species like *Erysipelothrix rhusopathiae*, *Lactococcus*, *Pediococcus*, *Lactobacillus*, and so forth, which are intrinsically resistant to the glycopeptide, the current paper focusses on the problem of acquired glycopeptide resistance in gram-positive cocci.

## 2. Vancomycin Resistance in Enterococci

The mechanism by which vancomycin exerts its action is by preventing the synthesis of peptidoglycan precursors of the bacterial cell wall by blocking the transglycosylation step and subsequently affecting the transpeptidation step also [3, 5]. Both the transglycosylation and transpeptidation steps are essential for bacterial cell wall cross-linking.

Vancomycin resistance in enterococci was first reported by Uttley et al. in 1988 from Great Britain [8]. Vancomycin-resistant enterococci (VRE) showing resistance to glycopeptides like vancomycin and teicoplanin have now been reported from many parts of the world and show heterogeneity, both phenotypic and genotypic [9]. There are as many as 6 recognized vancomycin-resistance phenotypes—VanA, VanB, VanC, VanD, VanE, and VanG [3, 10, 11]. Gene clusters corresponding to these phenotypes have been described. Recently, new gene clusters encoding for vancomycin resistance have been discovered (*vanL*, *vanM*, and *vanN*) [4, 6, 7].

### 2.1. Molecular Basis of Vancomycin Resistance in Enterococci

The basic mechanism of vancomycin resistance in enterococci is the formation of peptidoglycan receptors with reduced glycopeptide affinity. This results in decreased binding of vancomycin and decreased inhibition of cell wall synthesis. Peptidoglycan precursors with decreased binding to vancomycin are responsible for this. Instead of the normally occurring peptidoglycan precursor D-alanine-D-alanine, precursors like D-ala-D-lactate or D-ala-D-serine are found on the cell wall of vancomycin-resistant strains of enterococci. D-ala-D-lactate has been found to have an affinity 1000 times less than D-ala-D-ala for vancomycin whereas D-ala-D-serine has an affinity about 6 times less than the normal cell wall precursors. It has been shown that the substitution of the terminal D-alanine of the cell wall with D-lactate results in repulsive forces in the binding pocket of the vancomycin molecule leading to a 1000-fold decrease in affinity to the antibiotic [12]. D-ala-D-serine substitution leads to a 6-fold decrease in affinity to vancomycin because of the hydroxymethyl group of serine which is bulkier than the methyl group of alanine [13].

### 2.2. Phenotypes and Genotypes of Vancomycin-Resistant Enterococci

Table 1 depicts the various phenotypes of vancomycin resistance and the gene clusters associated with them.

VanA and VanB phenotypes of vancomycin resistance are characterized by high-level resistance to vancomycin (minimum inhibitory concentrations of 64–1000 *μ*g/mL) which is inducible in nature. VanA type, but not VanB type, strains also show high-level resistance to the other glycopeptide, teicoplanin. The inducing factors might be the previous use of glycopeptides like vancomycin in patients or the use of drugs like avoparcin and ristocetin in poultry [3]. The increased use of a glycopeptide, avoparcin, as a growth promoter in farm animals in Europe was found to be responsible for emergence of vancomycin resistant enterococci on farms making the farm animals a potential reservoir of infection for VRE in humans [14]. Avoparcin was therefore banned from the European Union in 1997 and studies have shown an attributable reduction in vancomycin resistance in enterococci isolated from human faecal carriers after the ban [15]. The other phenotypes which show inducible vancomycin resistance are VanG and VanE where the degree of resistance is low and the minimum inhibitory concentrations range from 8 to 32 *μ*g/ml [5]. VanD resistance phenotype is characterized by moderate- to high-level vancomycin resistance and low-level teicoplanin resistance and this resistance is also inducible in nature [16].

The other type of glycopeptide resistance seen in enterococci is constitutive, noninducible intrinsic low-level resistance, the VanC1/C2/C3 phenotypes. VanC resistance phenotype is seen in motile species of *Enterococcus *like *Enterococcus gallinarum* and *Enterococcus casseliflavus* [3]. The VanC ligase of these motile strains leads to the production of D-ala-D-serine in the terminal part of the bacterial cell wall pentapeptide.

A recently discovered new gene cluster, vanN, has also been found to confer a constitutive glycopeptide resistance phenotype [4].

### 2.3. Vancomycin Dependence in Enterococci

Vancomycin dependence is an interesting phenomenon seen in some strains of *Enterococcus *species for which growth is seen only in the presence of vancomycin as in vancomycin containing media or in patients on vancomycin therapy. The mechanism proposed for this is that vancomycin-dependent enterococci might lack a functional D-alanine-D-alanine ligase and are probably able to synthesize cell walls only from D-alanine-D-lactate precursors which are formed only in the presence of vancomycin [17].

### 2.4. Genetic Basis and Regulation of Vancomycin Resistance

The *vanA* operon responsible for the VanA type of high-level glycopeptides resistance was first found to be carried by a transposon Tn1546. This genetic element might be carried on plasmids or might be located on chromosomes [18]. The *vanA* and the *vanB* operons have been the most extensively studied of the vancomycin-resistance gene clusters. The *vanA* and *vanB* gene clusters have three major genes, *vanHAX* and *vanH*_*B*_ *BX*_*B*_. These genes encode for the following proteins essential for conferring glycopeptide resistance—a dehydrogenase (VanH/VanH_B_), a ligase (VanA/VanB), and a dipeptidase (VanX/VanX_B_). The dehydrogenase reduces pyruvate to D-Lactate, the ligase synthesizes D-alanine-D-lactate that is responsible for glycopeptide resistance, and the dipeptidase hydrolyses D-alanine-D-alanine precursors [19].

### 2.5. Testing for Vancomycin Resistance in Enterococci

According to the Clinical and Laboratory Standards Institute (CLSI), following are the MIC interpretative criteria for vancomycin for enterococci [20]:

- susceptible ≤4 *μ*g/ml,
- intermediate-8–16 *μ*g/ml,
- resistant ≥32 *μ*g/ml.

Vancomycin screen agar is a convenient way of screening for vancomycin resistance in busy clinical microbiology labs. Vancomycin screening agar was first described by Willey et al. [21] and incorporated in the CLSI guidelines in 1993. Vancomycin screen agar plates are prepared with brain heart infusion (BHI) agar and supplemented with 6 *μ*g/ml of vancomycin [22].

Using BHI agar instead of Mueller-Hinton agar (MHA) as the base has been proved to have a greater sensitivity and specificity [23]. Also, it has been observed that growth is sparser on MHA, thus sometimes making the interpretation of results difficult. Using 6 *μ*g/ml of vancomycin in BHI agar has been demonstrated to show 96–99% sensitivity and 100% specificity [24]. While looking for vancomycin resistance by vancomycin screening agar, it is important to use positive and negative controls. For quality control, *E*. *faecalis* ATCC 29212 should be tested as the negative control and *E*. *faecalis* ATCC 51299 should be tested as the positive control [25].

For studying the MIC of vancomycin in enterococci, agar microdilution with MHA as the base is recommended with the various values of MICs corresponding to sensitive, resistant, and intermediate as mentioned before [26]. *E*-tests are a convenient substitute to the agar dilution methods for studying MICs, but the results should be interpreted carefully.

Newer methods of detection of VRE include automated culture and identification systems and chromogenic media. In the beginning, automated systems like the Vitek and MicroScan systems had problems in detecting low-level vancomycin resistance in intrinsically resistant *Enterococcus* species [22]. However, newer Vitek 2 and Phoenix systems have been shown to perform quite well in detecting vancomycin resistance in these organisms. In a study by Carroll et al. which evaluated the capability of the BD Phoenix Automated Microbiology System to detect vancomycin resistant strains, all vancomycin-resistant strains were correctly identified by the system [27]. The most commonly used chromogenic selective medium for screening of GRE is the bile esculin azide agar supplemented with 6–8 *μ*g/ml of vancomycin. Until recently, it was the most commonly available screening agar also. Highly specific chromogenic substrates have been recently used to make the CHROM ID VRE (bioMe'reiux) and the CHROMagar GRE (BD Diagnostics). CHROM ID has chromogens which are targeted by enzymes present specifically in *E*. *faecalis* or *E*. *faecium.* The degradation of these substrates leads to the species forming purple and blue-green colonies, respectively [28]. In one study in 2008, the sensitivity and specificity of the CHROM ID agar has been evaluated to be 96.9% and 99.4%, respectively [28].

Molecular methods which detect the resistance genes responsible for resistance or decreased susceptibility to antimicrobial agents have the advantage of being rapid. These also play a major role in the understanding of the spread and genetics of enterococcal antimicrobial resistance [29]. However, since these are highly specific methods, they do not detect antimicrobial resistance due to mechanisms which are not targeted by the testing [29]. PCR protocols to directly identify VRE from stool samples have been developed and evaluated [30]. These are very helpful for surveillance of VRE using rectal swabs and stool samples and are less time consuming. Once standardized, these are also less expensive than the traditional culture screening methods. Since there are many genotypes of glycopeptide resistance, a multiplex PCR can prove helpful to detect which of the van genotypes is present in a particular isolate. One of the earliest multiplex PCR standardized to detect some of the resistance genes was by Dutka-Malen et al. in 1995 [31]. Many modifications to the PCR protocol used by Dutka-Malen et al. have been used, both in the primer sequences used and in the DNA extraction protocols.

In a study by Patel et al. in 1997, *Enterococcus* colonies were directly put in the PCR mixture. This particular study also used RFLP for differentiating the resistance genotypes [32]. Depardieu et al. optimized a multiplex PCR assay for detecting the genotypes of vancomycin resistance along with the species identification of *E*. *faecalis*, *E*. *faecium*, *S*. *aureus*, and *S*. *epidermidis* [33]. Real-time PCR has also been used for rapid identification and characterization of vancomycin-resistant enterococci [34].

### 2.6. Antimicrobial Agents Effective against VRE and Emerging Resistance

Linezolid is a member of a class of synthetic antimicrobials, the oxazolidinones. Although it is more expensive than vancomycin, linezolid has the advantage of not requiring testing for adequate serum drug concentrations or dose adjustment in renal or hepatic failure. It is therefore a valuable drug in the hands of clinicians and can be used in situations where vancomycin use is either contraindicated or ineffective. Linezolid was licensed for clinical use in the United States in 2000. It was approved for use in United Kingdom a year later. The first isolates of Enterococcus resistant to Linezolid were reported from the United Kingdom in 2002 [35]. Two of the isolates were *E*. *faecium*; one was *E*. *faecalis*. All the isolates were from patients who had been previously treated with Linezolid. Pulsed field gel electrophoresis (PFGE) analysis of the isolates indicated that resistance developed in previously susceptible strains via point mutations in the 23SrRNA [35]. Linezolid-resistant and vancomycin-resistant enterococci have also been isolated from patients without any prior therapy with Linezolid [36].

Kainer et al. performed a case-control study during a hospital outbreak of linezolid-resistant enterococci (LRE) and tried to find out putative risk factors for acquiring this infection [37]. Important risk factors which came up in the study were culture positive for MRSA, increased hospitalization duration before index culture and duration of preceding linezolid therapy [37].

Quinupristin-Dalfopristin (Q/D) is a semisynthetic antimicrobial which is administered parenterally. It belongs to a group of agents called Streptogramins. This drug has a broad spectrum of in vitro activity against gram-positive bacteria like MRSA, coagulase-negative Staphylococci, and MDR strains of *Streptococcus pneumoniae* [38]. It has also been found to be very effective against vancomycin strains of *E*. *faecium* [39], although against *E*. *faecalis*, it has been found to be bacteriostatic rather than bacteriocidal [40]. In a study by Winston et al., quinupristin-dalfopristin has been found to be effective in 86% of the cases of VRE in which it was used [38]. This is a well-tolerated drug apart from arthralgias or myalgias seen in higher doses in some patients. 

Resistance to Dalfopristin-Quinupristin has however already emerged. In a study done in the United States by Angulo et al. on *Enterococcus *isolates from poultry products and human faecal samples, Q/D-resistant *E*. *faecium* was isolated in a considerable proportion [41]. The use of virginiamycin, which is also a related streptogramin and is used in poultry as a growth promoter in Europe, has most likely led to the emergence of Q/D resistance there [41].

A study from UK by Johnson et al. also showed the emergence of Q/D resistance in a few strains of* E. faecium* along with most of the strains of *E*. *faecalis*, *E*. *casseliflavus*, and *E*. *gallinarum* [42].

Daptomycin is a cyclic lipopeptide with rapid bactericidal activity against a wide spectrum of gram-positive bacteria. Daptomycin has been used to treat complicated skin and soft tissue infections caused by *S. aureus* and also for treating enterococcal infections [43]. Large-scale in vitro studies have shown that daptomycin is effective against more than 98% of enterococci tested, irrespective of their susceptibility to other agents [43].

Resistance breakpoints for daptomycin have not been defined either by the CLSI or by the EUCAST. According to the CLSI, enterococcal isolates with MIC ≤4 *μ*g/ml are considered sensitive to daptomycin. Disk diffusion tests for daptomycin resistance are not defined by the CLSI. For MIC testing, the recommended methods are using daptomycin *E*-test strips with Ca^2+^ ion (40 *μ*g/ml) adjusted MHA and broth microdilution.

A study carried out by the daptomycin study group in India demonstrated that 100% of the VRE strains were susceptible to the agent. With regards to the VSE strains however, the potency of both daptomycin and vancomycin was comparable. 90% of the *E*. *faecium* strains tested in this study were susceptible to daptomycin [44].

Tigecycline is a broad-spectrum glycylcycline antimicrobial agent which was introduced in 2005. It is a tetracycline derivative which has in vitro activity against VRE. However, clinical data regarding the efficacy of this antibiotic is lacking [45, 46].

## 3. Vancomycin Resistance in *S. aureus*

### 3.1. Vancomycin Intermediate *S*. *aureus* (VISA)

Increasing prevalence of MRSA infections has led to the extensive use of vancomycin for treating these conditions. Infact, vancomycin is the treatment of choice for MRSA infections. However, the overuse of this antibiotic has led to the emergence of *S. aureus* strains with reduced susceptibility to vancomycin. Hiramatsu et al. from Japan were the first to report a clinical strain of methicillin-resistant *S*. *aureus* with reduced susceptibility to vancomycin. This strain, named Mu50, was isolated from pus from the sternal incision site of a 4-month-old male infant with pulmonary atresia [2]. Such strains have now been reported from many other countries. Tiwari and Sen were the first to report strains of *S*. *aureus* with reduced susceptibility to vancomycin (VISA) from the Indian subcontinent. Strains of *S*. *aureus* in the intermediate range of vancomycin susceptibility have also been reported from the southern part of India [47].

The concentration of vancomycin required to inhibit most strains of *S*. *aureus* ranges from 0.5 to 2 *μ*g/ml. According to the current guidelines given by the Clinical Laboratory Standards Institute (CLSI), *S*. *aureus* isolates with vancomycin MICs between 4 and 8 *μ*g/ml are classified as vancomycin intermediate and those with MIC ≥16 *μ*g/ml as vancomycin resistant. These MIC cut-offs are different from those for coagulase negative staphylococci and enterococci where isolates with vancomycin MIC ≥32 *μ*g/ml are classified as vancomycin resistant [20]. Apart from vancomycin intermediate *S*. *aureus* (VISA) and vancomycin-resistant *S*. *aureus* (VRSA), there is one more entity, heterogenous VISA (hVISA), which is a source of some confusion. Like VISA, heterogenous VISA was first reported from Japan, from the sputum of a 64-year-old man suffering from MRSA pneumonia who had been on vancomycin therapy. The strain named Mu3 when grown on drug-free medium produced subpopulations with varying degrees of vancomycin resistance [48]. hVISA has a vancomycin MIC ≤2 *μ*g/ml by routine testing methods but has a population of cells with reduced vancomycin susceptibility (in the vancomycin intermediate range) [49].

Unlike VRSA isolates, strains of VISA or hVISA do not possess vancomycin resistant genes like *vanA*, *vanB*, or *vanC*. Although no mechanism has yet been conclusively established for VISA or hVISA, many mechanisms have been proposed like defects in DNA mismatch repair [50]. The acquisition of VISA phenotype is probably a multiple step event and is due to changes in the process of peptidoglycan synthesis. VISA strains have been found to synthesise excess amounts of D-alanine-D-alanine. The extra layers of cell wall precursors prevent vancomycin molecules from reaching their target sites [48].

One important difference between VRSA and heterogenous VISA is that reduction in glycopeptide selective pressure in an environment might reduce VRSA prevalence. However, heterogenous VISA has been found to disseminate even in the absence of glycopeptide pressure [49].

### 3.2. Vancomycin-Resistant *S*. *aureus* (VRSA)

Ever since the discovery of vancomycin-resistant enterococcus in the late 1980s, concern regarding the emergence of vancomycin resistance in isolates of methicillin-resistant *S*. *aureus* by transfer of plasmids was there. A fully resistant strain of vancomycin-resistant *S*. *aureus*, however, emerged only in 2002 and was first reported from the United States of America [51]. These strains, as expected, had acquired the vancomycin resistance gene cluster vanA from vancomycin-resistant enterococci. Till date, 13 cases of VRSA infection have been reported, with the majority of the cases from the United States, that too from a particular area of the country, that is, Michigan (7 of the 11 isolates from the United States were from the Michigan area) [49, 52]. The other two isolates of VRSA were from Kolkata, India, and Tehran, Iran. For 10 out of the 11 strains of VRSA reported till date, acquisition of resistance plasmids has been found to be the responsible mechanism for vancomycin resistance. For the isolate of VRSA from Tehran, the genetic basis of vancomycin resistance has not yet been established. A recently published study in 2011 from Kolkata, India, reported yet another VRSA isolate from the Indian subcontinent [53]. Vancomycin resistance has been demonstrated to be inducible in vitro for many of the VRSA strains [54]. Also, in a majority of the cases, VRE strains have been isolated along with the VRSA strains from the same patients. This goes in favour of the popular theory that the Tn1546 plasmid which carries the vanA gene cluster found in such strains is acquired from VRE [52, 55]. 

### 3.3. Special Considerations in Detecting Vancomycin Resistance in *S. aureus*

#### 3.3.1. Changing CLSI Guidelines for VRSA

The susceptibility and resistance breakpoints for both minimum inhibitory concentration (MIC) and disk diffusion testing of vancomycin have been changed over time due to clinical and microbiological data which suggested that infections with strains of MRSA with vancomycin MICs more than 4 *μ*g/ml lead to treatment failure with vancomycin. In 2006, the susceptible breakpoint for vancomycin was lowered to 2 *μ*g/ml and the resistance breakpoint was changed to ≥16 *μ*g/ml [56].

#### 3.3.2. Testing for Heterogenous Vancomycin Intermediate S. aureus

Because the usual phenotypic tests cannot detect hVISA and there are no established genetic markers for vancomycin resistance in hVISA or VISA isolates, the detection of hVISA is quite difficult. The gold standard method for detection of vancomycin resistance in hVISA isolates is population analysis profile (PAP). Modifications of the PAP method where area under the curve (AUC) of the standard PAP graph is measured for the test strain and the hVISA reference strain Mu3 have been used in many studies for detecting hVISA. hVISA has been defined to have a PAP/AUC ratio of ≥0.9 [57]. Because it is not possible to carry out population analysis profile for all strains of MRSA, alternatives like the Macromethod *E*-test and *E*-test GRD have been evaluated. Varying sensitivity rates have been reported for these methods in many studies. The GRD E-test has been found to detect 71% of hVISA isolates in one study from Australia [49]. A similar study by Riederer et al. from Michigan, USA, also gave comparable results [58]. Another study from the United States gave out a much lower detection rate of only 57% by using the GRD *E*-test [49].

#### 3.3.3. Resistance Phenotypes of VRSA

Depending on the degree of resistance to the glycopeptides, vancomycin, and teicoplanin, two resistance phenotypes have been defined for VRSA isolates. VRSA strains with high-level resistance to both vancomycin and teicoplanin (MIC >256 *μ*g/ml and >32 *μ*g/ml) are named high-level resistant VRSA (HLR VRSA). Most of the isolates till date have HLR vancomycin resistance. Only two of the VRSA isolates had a moderate level of resistance to vancomycin (MIC 32 *μ*g/ml and 64 *μ*g/ml) and low-level resistance to teicoplanin and were designated low-level-resistant VRSA (LLR VRSA) [52].

### 3.4. Treatment Options for VRSA Infections

One interesting finding that has emerged from *in vitro* studies and animal studies done for VRSA isolates is that vancomycin and teicoplanin show a synergistic action with *β*-lactams against such strains [59]. However, whether this correlates with the clinical response in patients is yet to be seen. Linezolid is one treatment option for VRSA infection which has been found to have either a synergistic or additive effect on VRSA strains when combined with ampicillin/sulbactam [60].

Daptomycin is a lipopeptide which targets the bacterial plasma membrane and causes depolarization of the membrane and loss of membrane potential. This has been used for treatment of VRSA infections, but contrary to expectations studies have found a strong positive correlation between reduced daptomycin susceptibility and vancomycin-resistance in VISA isolates [61]. Among the other antimicrobials which show in vitro activity against vancomycin intermediate and vancomycin-resistant *S*. *aureus* are tigecycline which is a member of the tetracycline family and lipoglycopeptides like telavancin and oritavancin [49]. Newly developed cephalosporins like ceftaroline and ceftobiprole which have action against MRSA have also shown good *in vitro* activity against VISA and VRSA strains [62]. Whether any of these new antimicrobials emerge as viable alternatives for treatment of vancomycin nonsusceptible strains of *S*. *aureus*, however, is yet to be seen.

## 4. Vancomycin Resistance in ****Coagulase-Negative Staphylococci

Coagulase-negative staphylococci are increasingly becoming important causes of nosocomial infections. Although these are perhaps the commonest isolates from clinical samples in any diagnostic bacteriology laboratory, it is in many cases difficult to assign clinical significance to them as they are also normal commensals found on the skin surface and elsewhere. In 1987 the first clinically significant isolate of a coagulase-negative staphylococci showing resistance to vancomycin was described by Schwalbe et al. [63]. Since then, there have been many reports of clinically relevant isolates of coagulase negative staphylococci showing resistance to glycopeptides. Among the various species of coagulase-negative staphylococci (CoNS), *Staphylococcus haemolyticus* is the most common species associated with glycopeptide resistance [64]. Other species which have been associated with vancomycin resistance include *S*. *epidermidis*, *Staphylococcus warneri*, and *Staphylococcus hominis* [65]. In some studies, *S*. *epidermidis* has been found to be the commonest CoNS species associated with glycopeptide resistance [66, 67]. The prevalence of glycopeptide resistance among clinical isolates of CoNS has shown an upward trend in many studies worldwide [68].

Although the exact mechanism of glycopeptide resistance in coagulase-negative staphylococci has not yet been elucidated, glycopeptide- resistant strains of *S*. *epidermidis* and *S*. *haemolyticus* have been shown to differ considerably from glycopeptide-susceptible strains with respect to various parameters like cell wall composition and synthesis, binding to glycopeptides and even ultrastructural morphology [69]. Glycopeptide-resistant CoNS strains have been demonstrated to sequester glycopeptides like vancomycin and teicoplanin more efficiently than their glycopeptide sensitive counterparts at sites unassociated with the D-alanyl-D-alanine target [70]. Teicoplanin has been found to bind more avidly than vancomycin at these sites. Interestingly, there have been reports of strains of CoNS, especially *S*. *haemolyticus* which are resistant to teicoplanin, but vancomycin susceptible [71].

Like *S*. *aureus*, heterogenous resistance to glycopeptides has also been observed in coagulase-negative staphylococci. In some studies, population analysis profile (PAP) has been used to show the presence of populations of bacterial cells with raised glycopeptide MICs at significant frequencies (10^−4^–10^−5^) [72].

### 4.1. Detection of Vancomycin Resistance in Coagulase-Negative Staphylococci

The CLSI MIC breakpoints for coagulase negative staphylococci differ from those for *S*. *aureus*. According to the guidelines, any coagulase-negative *Staphylococcus* for which vancomycin MIC is ≥32 *μ*g/ml should be sent to a reference laboratory. For CoNS, isolates with vancomycin MIC ≤4*μ*g/ml are considered to be sensitive to the glycopeptide, whereas those with MIC ≥32 *μ*g/ml are classified as resistant to vancomycin [20].

Different methods have been evaluated for determining the vancomycin MIC for CoNS isolates. A few studies have demonstrated that the MICs obtained by *E*-test are 1-2-fold higher than those obtained by broth microdilution [73]. Automated susceptibility testing systems like Vitek 2 have also been found to give higher MIC values for vancomycin in case of CoNS isolates [74].

## 5. Vancomycin Tolerance in *Streptococcus pneumoniae*

Although vancomycin resistance is not known in *Streptococcus pneumoniae*, the phenomenon of vancomycin tolerance has been observed in a few strains. Though this phenomenon has also been described in some strains of *S*. *aureus*, it is with regards to *S*. *pneumoniae* that it is considered most alarming. Vancomycin tolerance has been defined as a minimum bactericidal concentration (MBC) 32-fold higher than the minimum inhibitory concentration (MIC) [75, 76]. Another definition used by workers in the field considers pneumococcal strains showing more than 1% survival after four hours of growth in the presence of vancomycin concentration more than 10 times the minimum inhibitory concentration as showing tolerance [77]. Vancomycin tolerance in *S*. *pneumoniae* has been linked to treatment failure in many cases. Vancomycin tolerant *S*. *pneumoniae* has been found to be difficult to eradicate in animal models of meningitis and clinical instances of treatment failure with vancomycin in cases of meningitis have also been reported [78]. Vancomycin tolerance in case of *S*. *pneumoniae* is important not only because it can lead to treatment failure but also because tolerance is considered a precursor phenotype to resistance [79]. The first strain of vancomycin tolerant *S. pneumoniae* to be isolated from the CSF of a patient with meningitis was named the Tupelo strain after the local hospital where the patient was admitted. McCullers et al. carried out studies on this strain to elucidate the mechanism behind the vancomycin tolerance. Their findings suggested a defect in the pathway which controls the phenomenon of autolysis in case of *S*. *pneumoniae*. Such a defect prevents the lysis of bacterial cell, in the presence of not only vancomycin but also other cell wall acting agents like penicillin and cephalosporins [78]. Novak et al. showed that the loss of function of one of the enzymes involved in a two-component sensor regulator system produces tolerance to vancomycin and other groups of antibiotics [79]. Experimental meningitis in rabbit models by lab mutants carrying mutations which affect the function of this enzyme (histidine kinase/phosphatase) failed to respond to vancomycin. Recent studies have shown that vancomycin tolerance also requires the presence of a mutated capsular polysaccharide apart from the defects in the autolysin pathway [80]. Vancomycin tolerance among clinical strains of *S*. *pneumoniae* has been reported from different parts of the world like the United States of America, Hong Kong, Columbia, and Republic of Korea [78, 81–83]. According to other studies, however, tolerance to vancomycin is not yet a major clinical problem. Many studies have failed to detect vancomycin tolerance among their strains of *S*. *pneumoniae* [84, 85]. However, it is essential to try to detect vancomycin tolerance in *S*. *pneumoniae* as such strains could herald the onset of resistance to vancomycin in this important pathogen.

## 6. Conclusion

Vancomycin has been the drug of choice for serious beta-lactam-resistant gram-positive infections for over three decades now. However, the emergence and spread of resistance to this glycopeptide as well as other glycopeptide agents like teicoplanin among clinically important gram-positive cocci like *Enterococcus* species, *S. aureus*, and coagulase-negative staphylococci has made it difficult to manage serious infections caused by such pathogens. Fortunately, vancomycin resistance has not yet emerged in some important pathogens like *Streptococcus pyogenes*, *Streptococcus agalactiae*, and *Streptococcus pneumoniae*. The existence of vancomycin tolerant strains of *S*. *pneumoniae*, however, is a cause of concern. It is important to look for alternatives to vancomycin and other glycopeptides in the treatment of serious gram-positive infections. It is also equally important to prevent the spread and emergence of glycopeptide resistance by taking proper infection control measures.

## Figures and Tables

**Table 1:** The “Van Alphabet” (Phenotypes and Genotypes of vancomycin resistant enterococci).

Phenotype	Genotype (Gene clusters)	Vancomycin Resistance	Teicoplanin Resistance	Type of resistance
VanA [3] (commonly in *E. faecalis* and *E. faecium*)	*vanA* gene cluster	High-level resistance MIC-64 *μ*g/mL- ≥ 1000 *μ*g/mL	High-level resistance MIC-16–512 *μ*/mL	High level inducible resistance
VanB [3] (commonly in *E. faecalis* and *E. faecium*)	*vanB* gene cluster	High-level resistance MIC-4–512 *μ*g/mL	Sensitive MIC ≤ 0.5 *μ*g/mL	High level inducible resistance
VanC [3] (*E. gallinarum*, *E. casseliflavus*, *E. flavescens*)	*vanC1*, *vanC2*, *vanC3* gene clusters	Low level resistance MIC-2 *μ*g/mL-32 *μ*g/mL	Sensitive MIC ≤ 0.5 *μ*g/mL	Low level constitutive resistance
VanD [4]	*vanD* gene cluster	Moderate-High level resistance MIC-64–256 *μ*g/mL	Low-level resistance MIC-4–32 *μ*g/mL	Inducible resistance
VanE [5]	*vanE* gene cluster	Low-level resistance MIC-16 *μ*g/mL	Sensitive MIC-≤0.5 *μ*g/mL	Inducible resistance
VanG [5]	*vanG* gene	Low level resistance MIC ≤16 *μ*g/mL	Sensitive MIC ≤ 0.5 *μ*g/mL	Inducible resistance
VanL [6]	*vanL* gene cluster	Low level resistance MIC-8 *μ*/mL	Sensitive	Inducible resistance
VanM [7]	*vanM*	High-level resistance MIC > 256 *μ*g/mL	High level resistance	Inducible resistance
VanN [4]	*vanN*	Low-level resistance MIC-16 *μ*g/mL	Sensitive MIC ≤ 0.5 *μ*g/mL	Constitutive resistance
